# Genetic counselors and the future of clinical genomics

**DOI:** 10.1186/gm565

**Published:** 2014-07-02

**Authors:** Barbara Bernhardt

**Affiliations:** 1 Hospital of the University of Pennsylvania, 3400 Spruce St, Penn Tower Room 1115, Philadelphia, PA 19104, USA

## Abstract

Barbara Bernhardt discusses how the increasing importance of genomics in the clinic will change the role of genetic counselors.

## Introduction

**Figure 1:**
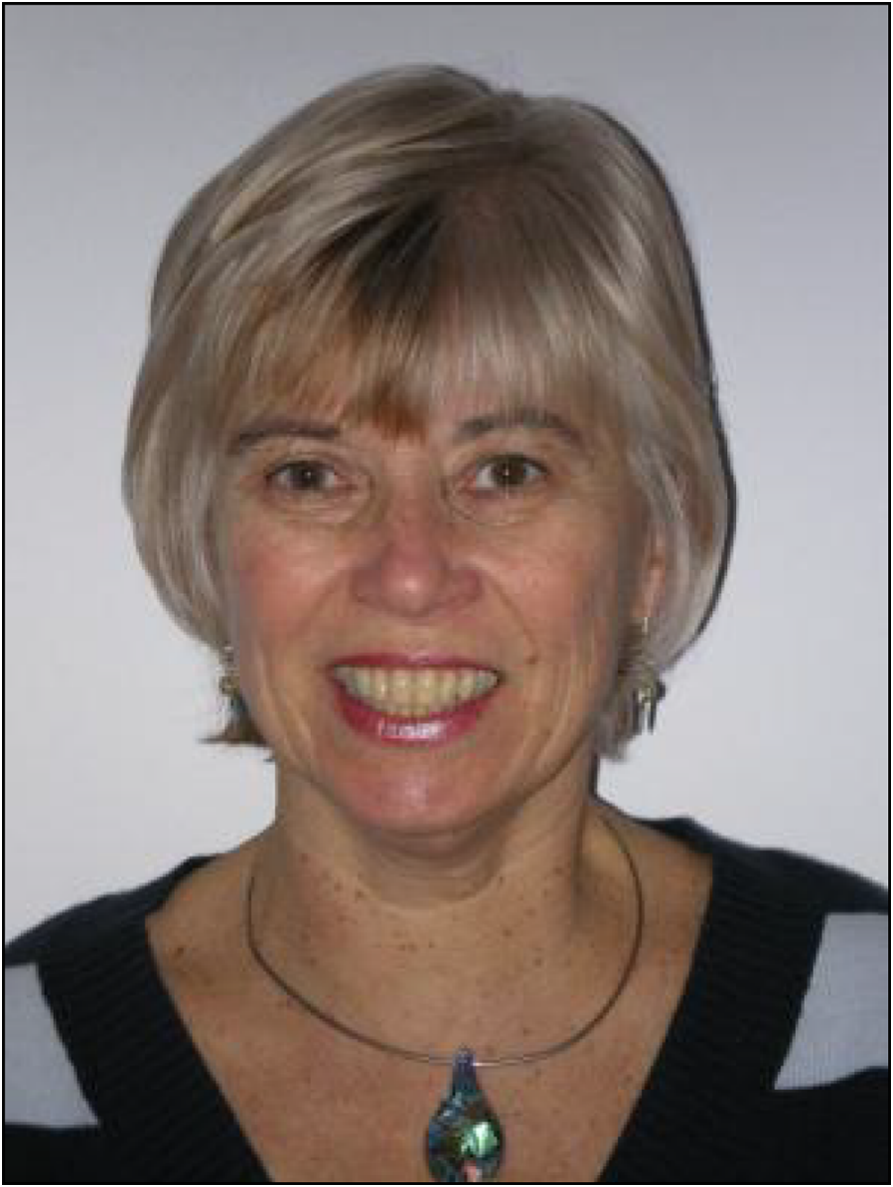
Barbara A Bernhardt

Barbara A Bernhardt, MS, CGC (Figure 1) is a genetic counselor and Clinical Professor of Medicine at the Hospital of the University of Pennsylvania, USA. She is also a social sciences researcher, currently conducting National Institutes of Health (NIH)-funded research on the utilization and outcomes of new genomic testing technologies such as genomic sequencing and chromosomal microarray testing. Here, she gives her views on the impact that the increasing use of genomics in clinical practice is having on genetic counselors, how patients are responding to increasingly complex information, and what the future of genetic counseling might look like.

## What does a genetic counselor do and how are genetic counselors trained?

Genetic counseling is ‘the process of helping people understand and adapt to the medical, psychological and familial implications of genetic contributions to disease’ [1]. Traditionally, genetic counselors have worked as members of a healthcare team, providing information and support to families who have members with birth defects or genetic disorders and to families who may be at risk for a variety of inherited conditions. Genetic counselors are employed in many settings, including medical centers, physician offices, health maintenance organizations, advocacy organizations, governmental agencies, public health departments and biotechnology companies. Those genetic counselors who work in clinical practice provide education and counseling in areas including reproductive, pediatric and adult genetics; newborn screening follow-up; and subspecialties such as oncology, cardiology and neurology. Many counselors also are actively involved in teaching and research.

In the United States and Canada, genetic counselors hold a masters degree from one of 35 accredited genetic counseling training programs. The didactic and clinical training components of these programs are aimed at supporting the development of competencies in four broad areas: genetics expertise and analysis; interpersonal, psychosocial and counseling skills; education; and professional development and practice. The current 3,500 trained genetic counselors in North America are board certified through the American Board of Genetic Counseling. In addition, there is currently licensure of genetic counselors in 14 US states.

## What recent advances in clinical genomics are impacting the work of genetic counselors?

In the past, genetic testing was used to look for variants in single genes that are associated with a specific condition, whose presence is suspected because of clinical presentation, family history or ethnicity. Recent technological advances, including next generation sequencing and genome-wide single nucleotide polymorphism (SNP) microarrays, have provided opportunities to test quickly, accurately and cheaply for variants in multiple genes simultaneously or for variants across the entire genome. These technologies have enabled new genomic applications in areas such as prenatal testing, carrier screening, newborn screening, diagnostic testing, predictive testing for common complex conditions, pre-symptomatic testing and pharmacogenetics [2]. In addition, the ability to test cell-free fetal DNA in maternal blood has led to rapid adoption of non-invasive prenatal testing for fetal aneuploidies and for some disorders associated with copy number variants. In the near future, rapid advances in bioinformatics, the increasing use of electronic medical records, and efforts aimed at integrating genome-wide data into these records will make patients’ genomic data immediately accessible for clinical use to aid in diagnosis, treatment and prevention.

As increasing amounts of genomic data become available, there will be a need to educate both clinicians and the public about the implications of the data for patients and their family members. These data will also raise important ethical questions relating to areas such as privacy and confidentiality, reproductive choices, and personal autonomy. Genetic counselors will be called upon to provide consultation for individual patients and clinicians to help with the interpretation of genomic data and to discuss options for acting upon the data.

## What new challenges are genetic counselors facing as a result of these changes?

The rapid advances in genomics will lead to an increase in the volume of test results and a need to understand the processes by which genetic variants are classified and interpreted.

Genetic counselors will be challenged to remain current in their knowledge of new technologies and their awareness of resources that are available to evaluate the implications of genomic variants.

The development of new genomic tests and therapies has led to high public and patient expectations for improved diagnostic capabilities and treatments. Tempering public expectations and educating patients about the limitations of genomic technologies will become an important role for genetic counselors, and will influence how we obtain informed consent and discuss test results with patients [3]. Because of the uncertainty surrounding the implications of much of the genomic variation that will be uncovered through genome-wide tests, genetic counselors increasingly will need to promote patient acceptance of genomic information with uncertain implications. In addition, genetic counselors will need to increase their own personal tolerance for uncertainty [4].

In the past, genetic counselors have had the luxury of spending an entire session discussing a patient’s susceptibility to and personal experiences with a single condition, such as breast cancer or cardiomyopathy. With the advent of genome-wide testing, many secondary findings may be available to be returned to patients, and genetic counselors will need to find new ways to streamline both pre-test education and counseling as well as post-test results disclosure. As some secondary findings are likely to be medically actionable, genetic counselors will need to gain expertise about the underpinnings of behavioral change as they increasingly engage with patients in health-promotion activities.

## How are patients and their families responding to the increasingly complex information that is being given to them?

Given the public’s low level of genetics literacy in general, genetic information has traditionally been viewed as complex. With the advent of genomic testing, the information being returned to families is becoming even more complex because there is more of it, much of it is probabilistic, and some has uncertain implications. Moreover, some of the information may be unanticipated and might relate to conditions of which families have no prior experience, as would be the case with incidental findings from whole-exome sequencing. Effective pre-test genetic counseling should prepare families for the possible disclosure of such information, and additional visits or other follow-up may be needed to promote optimal coping and understanding. But, as genomic testing becomes more routine, especially as it moves out of the genetics clinic and is offered by non-geneticist physicians, there may be less emphasis on pre-test counseling and post-test follow-up because of constraints on physician time. Families may respond by requesting genetic counseling or by turning to information available on the internet to help them make sense of genomic test results.

We’re just now beginning to gather data on how families understand and act on complicated genomic information. There is some anecdotal evidence that families may ascribe more importance to incidental findings than is warranted. In an attempt to make sense of uncertain information, families may also begin a search for additional information, or might choose to classify an uncertain finding as either pathogenic or benign [5]. Within the next few years, we will have data from various ongoing studies that have been funded by the National Institutes of Health and that are aimed at exploring outcomes, including psychosocial outcomes, of whole-exome and whole-genome sequencing.

## Is the relationship between genetic counselors, physicians and researchers changing?

As genomic testing diffuses into clinical care, I suspect that the expertise and contributions of genetic counselors will be increasingly recognized and valued. Genetic counselors will be the primary professionals called upon to educate primary-care providers and specialists, and to assist them with ordering genomic testing and with interpreting and understanding test results.

After genetic testing for susceptibility to breast and ovarian cancer became feasible in the mid-1990s genetic counselors were integrated into oncology services. As more genomic testing has become feasible for other groups of diseases, including neurologic, cardiologic and psychiatric diseases, genetic counseling services are being sought by these specialties. I would envision that, in the future, genetic counselors will be an integral part of many multidisciplinary teams. Because of this, genetic counselors will need to become familiar with the impact on patient management of results from a broader array of genetic tests.

As genomic research using human subjects expands, genetic counselors will be included in research teams to assist with obtaining informed consent and in returning results. Genetic counselors also contribute to research on the process of providing genomic services and assessing outcomes, especially those relating to genetic counseling.

## Are there enough genetic counselors?

Accurate data are not available to determine whether the current supply of genetic counselors is adequate to meet demand. Anecdotally, over the past few years, it appears that there are more positions available than there are trained counselors to fill them, although the number of training slots has increased about 25% over the past five years. In the near future, growth in the supply of genetic counselors will be limited by the number and size of training programs. In recognition of the need to increase the number of genetic counselors as the scope of practice expands, the National Society of Genetic Counselors has established a workforce growth working group to develop a strategic plan for increasing both the number and diversity of trained genetic counselors.

Increased hiring of genetic counselors has been tempered by inconsistent reimbursement for genetic counseling services resulting from a combination of factors, including lack of licensure, credentialing, insurance reimbursement and even billing for services provided. The National Society of Genetic Counselors has been addressing this issue and there has been gradual improvement; but it is probably correct to say that, at present, genetic counseling services are rarely self-sufficient.

## What do you foresee as the future of genetic counseling?

The future of genetic counseling will depend on how quickly and how broadly genomic testing technologies are incorporated into clinical care. This adoption in turn will be predicated on studies documenting that new genomic tests improve patient outcomes and reduce costs. In addition, implementation will be driven by patient and healthcare providers’ demand for the technologies, and payers’ willingness to reimburse for genomic testing and counseling.

In the past century, genetic counselors were primarily a part of a team, including a clinical geneticist, that provided services in a genetic clinic. Service-delivery models are likely to change as genomics is integrated into primary and specialty care. To improve access, telemedicine counseling opportunities will increase as will the need for e-learning approaches. Because most genetic counselors are drawn to the profession because of an opportunity to provide one-on-one counseling to families, either as a part of a team or independently, such services are likely to persist so long as families and providers find value in them. But in addition to serving individual patients and families, in the future, I envision that genetic counselors will be serving the provider community and the healthcare system by taking on the role of gatekeeper to genomic tests.

Moving forward, there will be many new and diverse opportunities for genetic counselors as researchers, consultants and educators. I see an incredibly exciting but somewhat uncertain future for genetic counseling. Because of our organization through the National Society of Genetic Counselors and through our alliances with other professional and lay organizations, we are well positioned to chart our own future course. 

## Competing interests

The author declares that she has no competing interests.
